# cVEMP correlated with imbalance in a mouse model of vestibular disorder

**DOI:** 10.1186/s12199-019-0794-8

**Published:** 2019-06-01

**Authors:** Reina Negishi-Oshino, Nobutaka Ohgami, Tingchao He, Kyoko Ohgami, Xiang Li, Masashi Kato

**Affiliations:** Department of Occupational and Environmental Health, Nagoya University Graduate School of Medicine, 65 Tsurumai-cho, Showa-ku, Nagoya, Aichi 466-8550 Japan

**Keywords:** cVEMP, Balance, Vestibule, Hair cells, IDPN

## Abstract

**Background:**

Cervical vestibular evoked myogenic potential (cVEMP) testing is a strong tool that enables objective determination of balance functions in humans. However, it remains unknown whether cVEMP correctly expresses vestibular disorder in mice.

**Objective:**

In this study, correlations of cVEMP with scores for balance-related behavior tests including rotarod, beam, and air-righting reflex tests were determined in ICR mice with vestibular disorder induced by 3,3′-iminodipropiontrile (IDPN) as a mouse model of vestibular disorder.

**Methods:**

Male ICR mice at 4 weeks of age were orally administered IDPN in saline (28 mmol/kg body weight) once. Rotarod, beam crossing, and air-righting reflex tests were performed before and 3–4 days after oral exposure one time to IDPN to determine balance functions. The saccule and utricles were labeled with fluorescein phalloidin. cVEMP measurements were performed for mice in the control and IDPN groups. Finally, the correlations between the scores of behavior tests and the amplitude or latency of cVEMP were determined with Spearman’s rank correlation coefficient. Two-tailed Student’s *t* test and Welch’s *t* test were used to determine a significant difference between the two groups. A difference with *p* < 0.05 was considered to indicate statistical significance.

**Results:**

After oral administration of IDPN at 28 mmol/kg, scores of the rotarod, beam, and air-righting reflex tests in the IDPN group were significantly lower than those in the control group. The numbers of hair cells in the saccule, utricle, and cupula were decreased in the IDPN group. cVEMP in the IDPN group was significantly decreased in amplitude and increased in latency compared to those in the control group. cVEMP amplitude had significant correlations with the numbers of hair cells as well as scores for all of the behavior tests in mice.

**Conclusions:**

This study demonstrated impaired cVEMP and correlations of cVEMP with imbalance determined by behavior tests in a mouse model of vestibular disorder.

**Electronic supplementary material:**

The online version of this article (10.1186/s12199-019-0794-8) contains supplementary material, which is available to authorized users.

## Introduction

Vestibular deficit causes vertigo and motion sickness and impairs motor functions. Vestibular evoked myogenic potential (VEMP) testing is a clinical examination for objectively determining vestibular functions in humans [1–3]. Cervical VEMP (cVEMP) and ocular VEMP (oVEMP) are clinically used to determine vestibular activities derived from the saccule and utricle in humans, respectively [1–3]. VEMP has been shown to significantly correlate with scores of posturography and beam walking in humans [4].

Behavior tests including rotarod, beam, and air-righting reflex tests have been used to objectively determine balance in a mouse model of vestibular disorder [5, 6]. The rotarod test is also used to determine balance and coordinate movement in mice with central nervous disorders including cerebellar ataxia [7], multiple system atrophy (MSA) [8], and parkinsonism [9]. To our knowledge, however, there is no information about impaired cVEMP in a mouse model of vestibular disorder, while only three previous studies have succeeded in showing impairments of cVEMP in guinea pigs treated with gentamicin [10–12]. Furthermore, the correlation of cVEMP with imbalance determined by behavior tests in experimental animals remains unclear.

In previous studies, experimental animals exposed to iminodipropionitrile (IDPN), which is a nitrile-related chemical and is a potential environmental factor affecting human health [13–15], were used as an animal model of vestibular disorder [16, 17]. In this study, wild-type mice were exposed orally to IDPN at a concentration of 28 mmol/kg and it was then examined whether cVEMP correlates with scores in imbalance behaviors determined by rotarod, beam, and air-righting reflex tests in the mouse model of vestibular disorder.

## Methods

### Mice

Male ICR mice purchased from Japan SLC (Hamamatsu, Japan) were randomly bred and used for experiments at 4–5 weeks of age. The mice were maintained under specific pathogen-free (SPF), constant temperature (23 ± 2 °C), and 12-h light/dark cycle conditions.

### Oral administration of IDPN

The method described in a previous report was used [18]. For the IDPN group, mice at 4 weeks of age were orally administered IDPN (Wako) in saline (28 mmol/kg body weight) once with a sonde (FTP-22-25, Prime Tech Ltd.). For the control group, mice at the same age were administered only saline in the same way. All behavioral tests and cVEMP testing were performed before and 3–5 days after IDPN administration.

### Behavior tests

The method reported previously was used for the rotarod test [19]. Mice (4 weeks of age) were placed on a stable rod of 5.7 cm in diameter (47600-Mouse Rota-Rod, UGO BASILE, Italy), and then the rod was rotated at a constant speed of 10 rpm/min for 10–20 s as training. The mice were then placed on the rod before and 3 days after administration of IDPN, and the rod was rotated at a constant speed of 25 rpm/min up to 300 s. Each trial was aborted if the mouse fell from the rod, and the latency until slipping on the rotarod was recorded. For the beam crossing test, mice were forced to walk on an elevated round wooden bar of 15 mm in diameter, and the time taken to traverse the bar of 30 cm in length was recorded twice up to 10 s. Before the test, mice walked on a round wooden bar of 22 mm in diameter once for training. Triplicated measurements were performed with 5-min intervals in each trial for the rotarod and beam crossing tests. For the air-righting reflex test, the method reported previously was used [20]. This test was performed after finishing the other behavior tests. A soft sponge was used to relieve the dropping impact. All animals were set in a supine position at 0.50 m in height and then dropped. The time it took for each animal to assume a prone position with all four legs in down in the air was determined. Each animal’s behavior was recorded with a high-speed camera (EXILIM EX-10, CASIO) three times.

### Immunohistochemistry

We performed perfusion fixation with 4% paraformaldehyde (PFA) for mice in the control and exposure groups 1–2 weeks after IDPN administration. After collection of inner ears, we further immersed the inner ears in EDTA for 2 days at 4 °C for decalcification. Paraffin sections of inner ears of 3 μm in thickness were prepared. Immunostaining with a polyclonal antibody against Myosin VIIa (1:100, Proteus BioScience) was performed after antigen retrieval had been performed with a TE buffer (pH 9.0) at 90–92 °C for 10 min. Alexa Fluor 594-labeled donkey anti-rabbit IgG (1:1000, Invitrogen) was used as a secondary antibody followed by counterstaining with 4′,6-diamidino-2-phenylindole (DAPI). The stained specimens were observed under a fluorescent microscope with phase contrast (Leica DM6000B). A researcher who was blinded to the results of cVEMP testing counted the numbers of hair cells in vestibular sensory epithelia. Hair cells were defined by the expression of Myosin VIIa in the cytoplasm and nuclear exclusion of the staining [21, 22].

### Phalloidin staining of vestibular hair cells

After treatment with IDPN, perfusion fixation with 4% paraformaldehyde (PFA) was performed. The utricles were isolated after dissection of inner ears. Hair bundles were labeled with fluorescein-phalloidin (Wako, 068–06261) diluted with PBS (1:200). The stained specimens were observed under a fluorescent microscope with phase contrast (Leica DM6000B).

### cVEMP measurement

cVEMP was recorded in mice by using methods established in previous studies [10, 12, 23]. Mice were first anesthetized under inhalation of isoflurane (Wako) to set platinum-needle electrodes in the neck extensors and a reference electrode in the occipital area. When we set up the measurement system for cVEMP, we pathologically confirmed the exact site of the neck extensor muscles after opening the targeting area in order to accurately place the needle electrodes in the neck extensor muscles. In addition, we always placed the electrodes at the same sites with reference to a position of the spinal cord at the level of C3. Electromyography (EMG) potentials were monitored to check whether mice were awake. After awaking, recording of cVEMP in EMG was started with an auditory brainstem response (ABR) system (Tucker-Davis Technologies, FL, USA) and PowerLab (AD Instruments Pty. Ltd) by stimulation with sound at 1000 Hz, SPL of 90 dB, rise-fall of 0.2 ms, interval of 200 ms, and 10-ms flat that was generated by a sound generator system (Sound Stimulator DPS-725, Dia Medical System CO., LTD, Japan). The sound stimulation was given by a speaker to the right ear of each mouse at a distance of 20 cm from the speaker. The onset was 0 ms After stimuli 200 times, integrated EMG showed similar potentials in the control group and IDPN group. During VEMP recording, the animal’s neck was stretched by lifting up the inhalation mask with polypropylene string to induce tonus of the sternocleidomastoid muscle. The inhalation mask was set at 2 cm in height from the ground (Additional file 1: Figure S1). All recordings were performed under wakefulness. We followed the definition of “normal” cVEMP in previous reports [12, 23, 24], which was the presence of a positive (p1)-negative (n1) waveform (i) at a 6- to 9-ms latency (ii) with a p1-n1 amplitude of 5 to 20 μV and (iii) the presence of a preceding negative wave just before cVEMP. In addition to this definition, we regarded the waveform as cVEMP when it appeared under the condition of (iv) stimulation of air-conducted sound at 1000 Hz and 90 dB (v) with the tension of the sternocleidomastoid muscle (SCM) by head tilt at 2 cm in height from the ground. We measured the background amplitudes under the condition of sound stimulation but not head tilt. We used the amplitudes “without head tilt” at the same latencies as those of cVEMPs obtained under the conditions of sound stimulation and head tilt. We normalized cVEMP amplitudes by subtracting the background amplitudes of the electromyography (EMG) potentials from the cVEMP amplitudes of cVEMP. We used the normalized cVEMP amplitudes for Fig. 3d and left graphs of Figs. 4 and 5. The results of comparisons of the control and IDPN groups (Fig. 3) and correlations (Figs. 4 and 5) were similar to the results without normalization.

### Statistics

Two-tailed Student’s *t* test was used to determine a significant difference between the two groups for parametric data [25]. Welch’s *t* test was also used to determine a significant difference between the two groups for parametric data with unequal variance. Spearman’s rank correlation coefficient and Spearman’s rank correlation coefficient were used to determine a significant correlation between nonparametric variables. A difference with *p* < 0.05 was considered to indicate statistical significance. All statistical analyses were performed using JMP Pro (version 13.0.0; SAS Institute Inc., Cary, NC, USA).

## Results

### Balance impairments of mice treated with IDPN

**Fig. 1:**
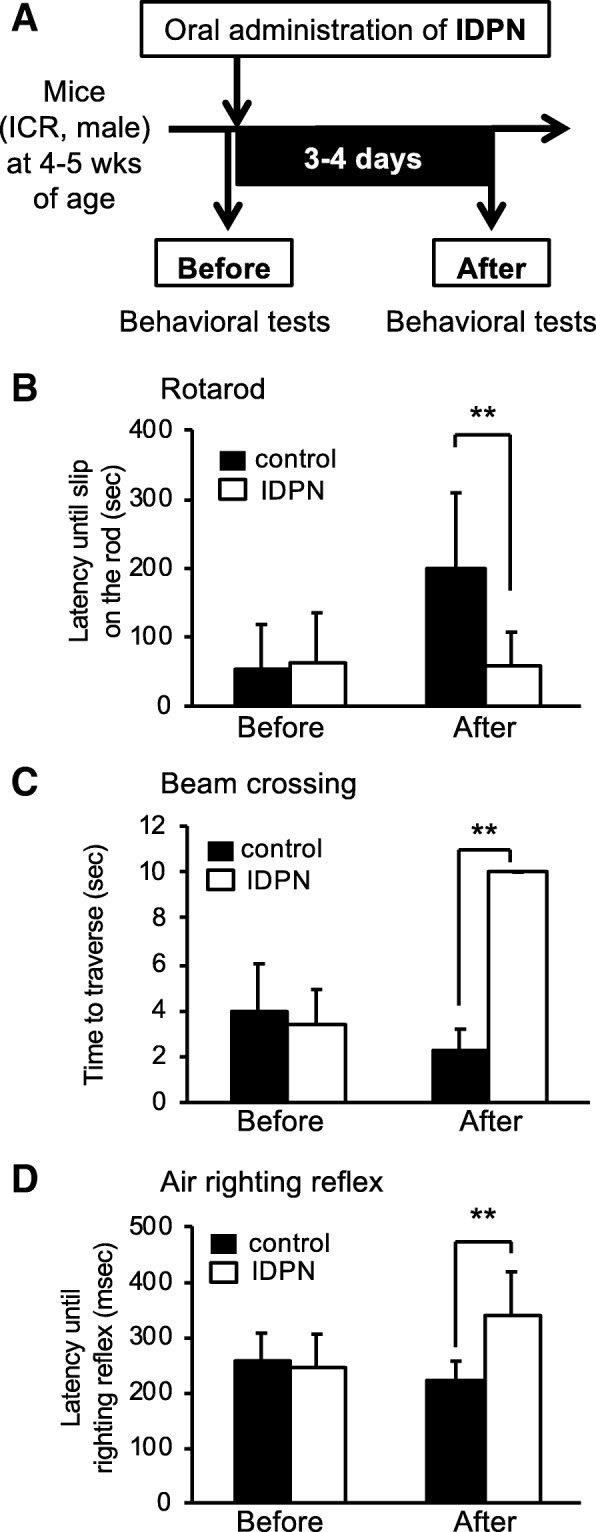
Balance impairments of mice treated with IDPN and a scheme of experiments. **a** Scheme of experiments. Behavioral tests were performed with 4–5 aged male mice following oral administration of IDPN. The control group (*n* = 5) and the IDPN group (*n* = 5) were subjected to behavioral tests after administration of IDPN. **b** A rotarod test, **c** a beam crossing test, and **d** an air-righting reflex test were performed before and 3–4 days after administration of IDPN (28 mmol/kg) to wild-type mice having an ICR background. **b** Latency until falling or slipping on the rotarod (seconds, mean ± SD), **c** time to traverse (seconds, mean ± SD), and **d** latency until righting reflex (milliseconds) were recorded for the control group (black bars, *n* = 5) and the IDPN group (white bars, *n* = 5). For the beam crossing test, a score of 10 s was given for mice that could not walk on the beam anymore because of complete loss of balance. Significant differences (**p < 0.01) between the two groups were analyzed by two-tailed Student’s *t* test

**Fig. 2:**
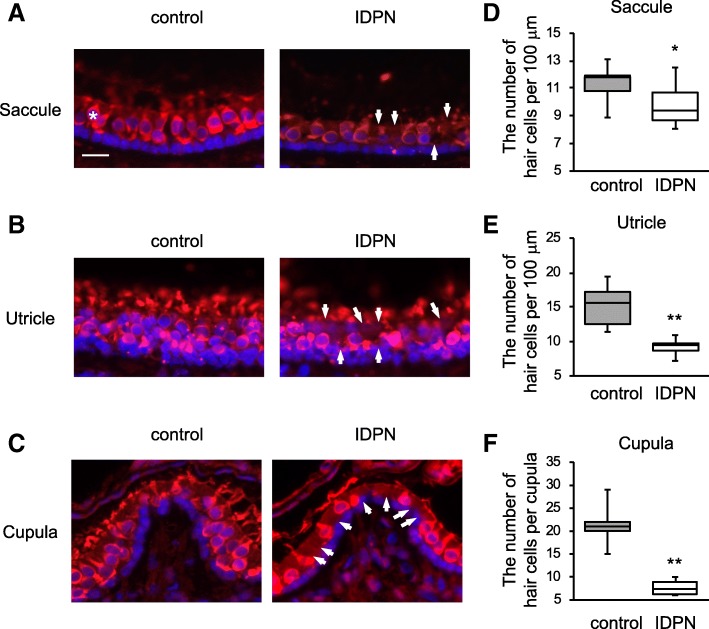
Vestibular hair cell loss in the saccule, utricle, and cupula of mice treated with IDPN. **a**–**c** After administration of IDPN, hair cells were stained with anti-MyosinVIIa antibody. Equivalent positions in the saccule (**a**), utricle (**b**), and cupula (**c**) from mice in the control group (left panels) and mice in the exposure group (right panels) are shown with the same scale (scale bar 20 μm in **a**). An asterisk shows an example of normal hair cells (**a**), and arrows indicate loss of hair cells (**a**–**c**, right panels). **d**, **e** Numbers of hair cells per 100 μm (mean ± SD) in the saccules (**d**) and utricles (**e**) from 3 mice in the control group (gray) and 3 mice in the IDPN group (white) are shown. **f** Numbers of hair cells in the cupula (means ± SD) from three mice in the control group (gray) and 3 mice in the IDPN group (white) are shown. Significant differences (*p < 0.05, **p < 0.01) between the two groups were analyzed by Welch’s *t* test

Rotarod, beam crossing, and air-righting reflex tests were performed before and 3–4 days after oral exposure one time to IDPN at 28 mmol/kg in order to determine balance functions in the control group and IDPN group. Before exposure to IDPN, the two groups showed comparable scores in the rotarod, beam crossing, and air-righting reflex tests (Fig. 1b–d). Latency until slipping on the rotarod in the IDPN group was significantly decreased compared to that in the control group (Fig. 1b). The time taken to traverse the beam in the IDPN group was significantly increased compared to that in the control group (Fig. 1c). Latency until air-righting reflex in the IDPN group was significantly increased compared to that in the control group (Fig. 1d). Next, immunohistostaining of vestibular hair cells from mice after oral exposure to IDPN for 1–2 weeks was performed to morphologically determine the influence of IDPN on vestibular hair cells. The numbers of hair cells in the saccule, utricle, and cupula from mice in the IDPN group were significantly decreased compared to those in the control group (Fig. 2). In phalloidin staining of vestibular hair cells from mice after oral exposure to IDPN, the numbers of hair bundles in the saccule and utricle from mice in the IDPN group were significantly decreased compared to those in the control group (Additional file 1: Figure S2).

### Impairments of cVEMP in the IDPN group

**Fig. 3:**
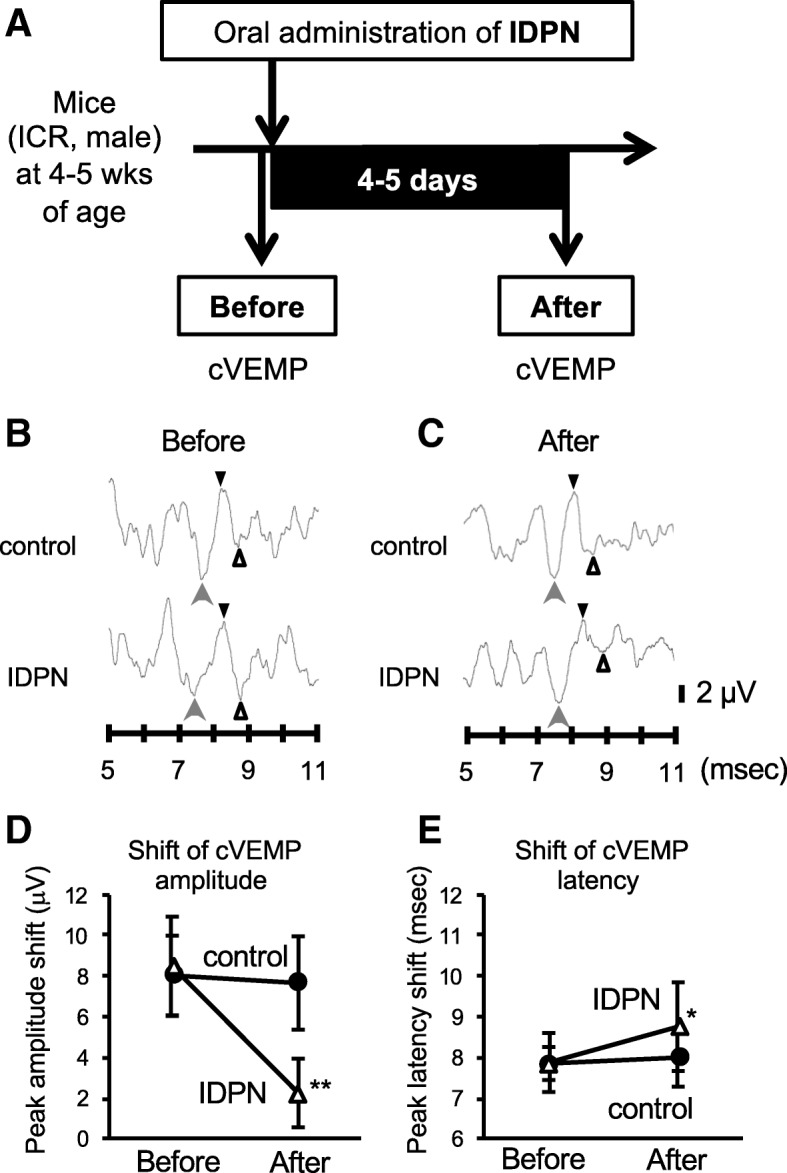
Impairments of cVEMP in the IDPN group and a scheme of experiments. **a** Scheme of experiments. cVEMP recordings were performed following oral administration of IDPN in male mice in the control group (*n* = 5) and the IDPN group (*n* = 5). cVEMP recording was performed after the administration of IDPN. **b**, **c** cVEMPs in the control group (upper waves) and the IDPN group (lower waves) before (**b**) and after administration of IDPN (**c**) are shown. cVEMPs with a positive peak (p1, indicated by black triangles) and those with a negative peak (n1, indicated by white triangles) were identified by the appearance of negative waves (indicated by gray arrows) just before p1 indicated by black triangles. **d**, **e** Graphs of cVEMP amplitudes [microvolts (μV), mean ± SD in **e**] and latencies [milliseconds (msec), mean ± SD in **e**] in the control group (black circles, *n* = 5) and the IDPN group (white triangles, *n* = 5) before and after administration of IDPN are presented. Significant differences (**p < 0.01; *p < 0.05) between the two groups were analyzed by Welch’s *t* test

At 4 days after the oral administration of IDPN, cVEMP measurements were performed for mice in the control and IDPN groups (Fig. 3). cVEMP waves in the control group (upper) and the IDPN group (lower) are presented at the same scale. The waveforms of cVEMPs were identified as cVEMPs with a positive peak (p1, indicated by black triangles) and those with a negative peak (n1, indicated by white triangles) by the appearance of preceding negative waves (indicated by gray arrows) just before p1 indicated by black triangles. cVEMPs in the two groups were comparable before exposure to IDPN (Fig. 3b). After administration of IDPN, the IDPN group showed decreased cVEMP amplitude and prolonged cVEMP latency compared to those in the control group (Fig. 3c). cVEMP amplitudes and latencies were compared in the control group and IDPN group (Fig. 3d, e). The amplitude in the IDPN group was significantly smaller than that in the control group, while the two groups showed comparable amplitudes before administration of IDPN (Fig. 3b–d). The p1 latencies in the IDPN group were significantly prolonged compared to those in the control group (Fig. 3e). We also determined the amplitudes of the preceding negative waves in the control and IDPN groups (Additional file 1: Figure S3). The amplitudes of the preceding negative waves were not significantly different in the two groups (Additional file 1: Figure S3A), while the amplitudes of the p1-n1 waveforms showed a significant difference in the two groups (Additional file 1: Figure S3B). ABR was also measured to confirm the cVEMP waveforms in mice. Typical waveforms of ABR at 20–80 dB of 4 kHz sound were detected in the control group but not in the IDPN group (Additional file 1: Figure S4) as previously reported [26, 27].

### Correlation between scores of the beam test and cVEMP amplitude

**Fig. 4:**
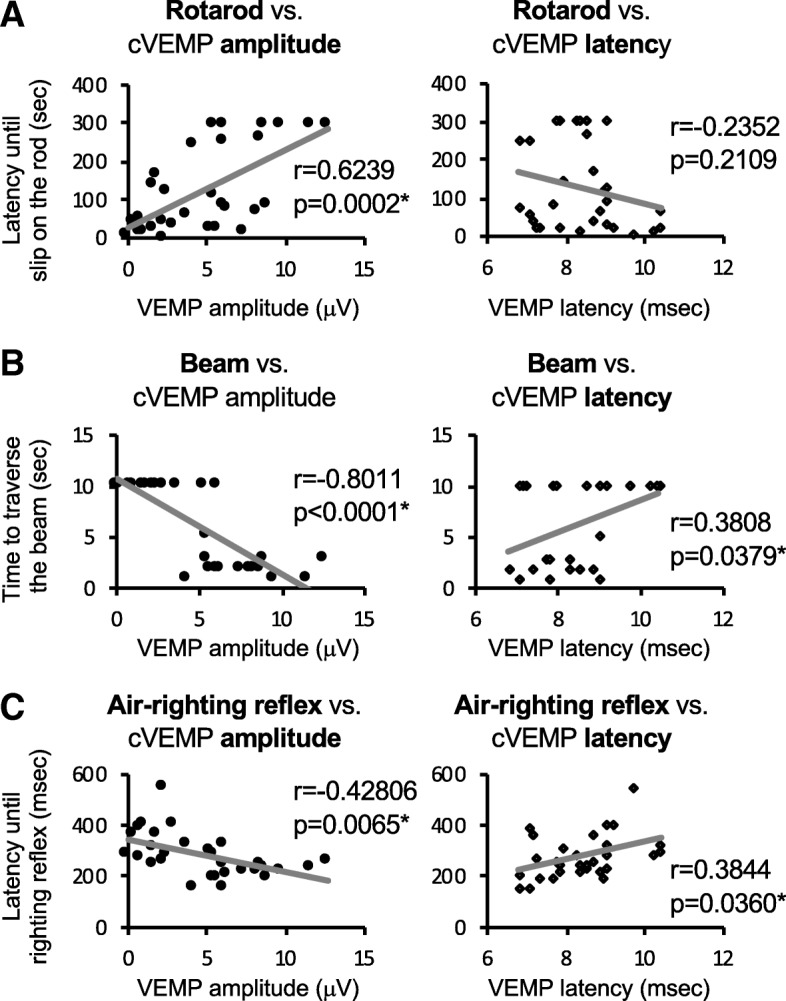
Correlations between scores of the behavior tests and cVEMP results. **a**–**c** Correlations of **a** rotarod test score [latency until slipping on the rod, seconds (sec)], **b** beam crossing test score [time to traverse the beam, seconds (sec)], and **c** air-righting reflex test score [latency until righting reflex, milliseconds (msec)] with cVEMP amplitude [microvolts (μV), left graphs] and VEMP latency (msec, right graphs) are shown. Triplicated measurements for each test with a total of 10 mice including 5 mice for the control group and 5 mice for the IDPN group were performed. Spearman’s rank correlation coefficients and significance differences (*p < 0.05) were analyzed for **a** rotarod vs. cVEMP amplitude [*r* = 0.6239, *p* = 0.0002] and cVEMP latency [r = − 0.2352, *p* = 0.2109], **b** beam vs. cVEMP amplitude [*r* = − 0.8011, *p* < 0.0001] and cVEMP latency [*r* = 0.3808, *p* = 0.0379], and **c** air-righting reflex vs. cVEMP amplitude [*r* = − 0.42806, *p* = 0.0065] and cVEMP latency [*r* = 0.3844, *p* = 0.0360]

The correlations between scores of behavior tests and amplitude or latency of cVEMP were determined (Fig. 4). There were significant correlations of time to traverse on the beam with cVEMP amplitude (*r* = − 0.8011, *p* < 0.0001) and latency (*r* = 0.3808, *p* = 0.0379) (Fig. 4b), and there were significant correlations of air-righting reflex with cVEMP amplitude (*r* = − 0.42806, *p* = 0.0065) and latency (*r* = 0.3844, *p* = 0.0360) (Fig. 4c). On the other hand, only the cVEMP amplitude had a significant correlation with the rotarod test score (*r* = 0.6239, *p* = 0.0002) (Fig. 4a).

### Correlation between number of hair cells and cVEMP amplitude

**Fig. 5:**
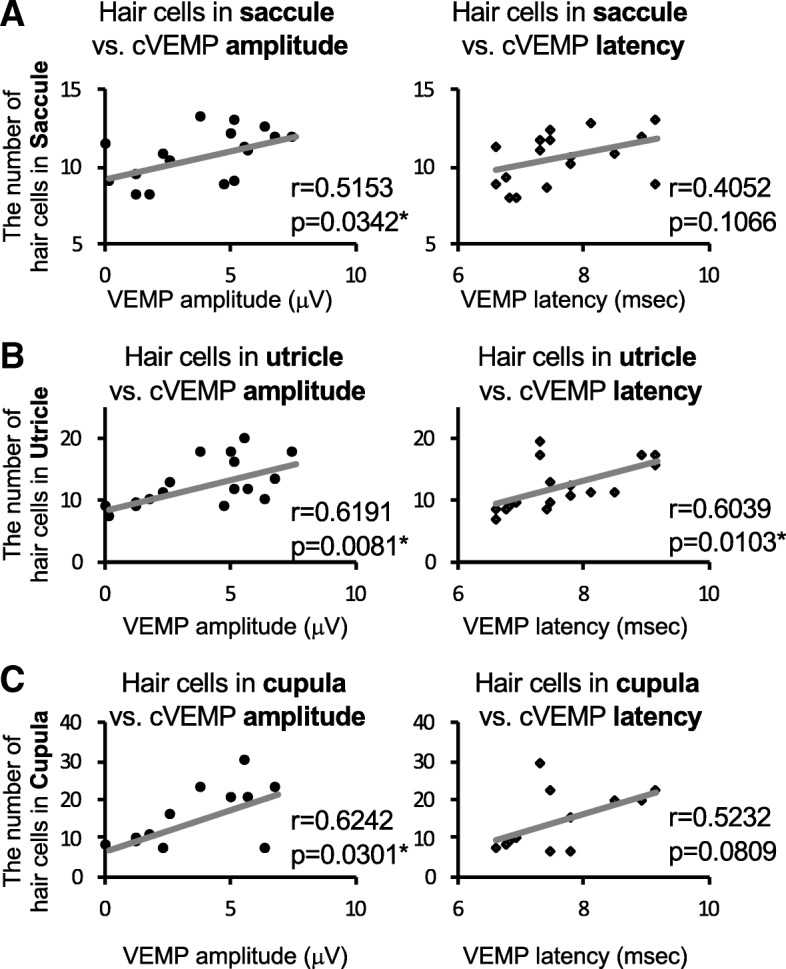
Correlations between the numbers of hair cells and cVEMP results. **a**–**c** Correlations of the numbers of hair cells in the saccule (**a**), utricle (**b**), and cupula (**c**) with cVEMP amplitude [microvolts (μV), left graphs] and VEMP latency (msec, right graphs) are shown. Triplicated measurements for cVEMP with a total of 6 mice including 3 mice for the control group and 3 mice for the IDPN group were performed. Spearman’s rank correlation coefficients and significance differences (*p < 0.05) were analyzed for **a** hair cells in the saccule vs. cVEMP amplitude [*r* = 0.5153, *p* = 0.0342] and cVEMP latency [*r* = 0.4052, *p* = 0.1066], **b** hair cells in the utricle vs. cVEMP amplitude [*r* = 0.6191, *p* = 0.0081] and cVEMP latency [*r* = 0.6039, *p* = 0.0103], and **c** hair cells in the cupula vs. cVEMP amplitude [*r* = 0.6242, *p* = 0.0301] and cVEMP latency [*r* = 0.5232, *p* = 0.0809]

Finally, correlations of the numbers of hair cells in the saccule, utricle, and cupula with the amplitude or latency of cVEMP were examined (Fig. 5) because associations of the utricle and cupula with cVEMP were previously reported [28, 29]. The number of hair cells in the saccule was significantly correlated with cVEMP amplitude (*r* = 0.5153, *p* = 0.0343) but not with cVEMP latency (*r* = 0.4052, *p* = 0.1066) (Fig. 5a), and there were significant correlations of the number of hair cells in the utricle with both cVEMP amplitude (*r* = 0.6191, *p* = 0.0081) and latency (*r* = 0.6039, *p* = 0.0103) (Fig. 5b). The number of hair cells in the cupula was significantly correlated with cVEMP amplitude (*r* = 0.6242, *p* = 0.0301) but not with cVEMP latency (*r* = 0.5232, *p* = 0.0809) (Fig. 5c).

## Discussion

In this study, the IDPN group showed imbalance behaviors. Rotarod, beam, and air-righting reflex tests were performed in this study since previous studies showed imbalance behavior in a mouse model of vestibular disorder [5, 6]. The IDPN group also showed decreased numbers of hair cells in the saccule, utricle, and cupula. IDPN is a nitrile-related chemical that is used in chemical industries for the manufacture of various products including plastics, pharmaceutical materials, fibers, and resins [30]. Exposure to the nitrile-related chemical has been shown to cause neuro-behavioral impairments in humans [31]. In experimental studies, oral exposure to IDPN has been shown to induce a vestibular disorder with a dose-dependent loss of vestibular hair cells and the crista ampullaris in the semicircular canals in rats, mice, and guinea pigs [18, 32] and imbalance behaviors in a rotarod test in mice [17]. Therefore, oral exposure of mice to IDPN was performed in this study to induce a mouse model of vestibular disorder.

cVEMP in mice with vestibular disorder was determined in this study since the establishment of cVEMP in mice will enable clarification of the molecular mechanism related to cVEMP with genetically engineered mice in future studies. This study demonstrated impairments of cVEMP in mice orally exposed to IDPN as an example of a mouse model of vestibular disorder. Our results suggest that cVEMP can be used to determine vestibular function in not only humans but also mice. We measured cVEMP under anesthesia with isoflurane to compare the detection of cVEMP under the condition of anesthesia and that under the condition of wakefulness (Additional file 1: Figure S5). cVEMP was not detected under the condition of anesthesia (Additional file 1: Figure S5A) but clearly detected under the condition of wakefulness (Additional file 1: Figure S5B). Thus, these results suggest that all of the recordings of cVEMP in this study were obtained under the condition of wakefulness in this study. In this study, we needed clearer waveforms of cVEMP in order to compare the amplitudes of cVEMP in the control and IDPN groups. In our comparison of cVEMPs with different intensities at 70, 80, and 90 dB of sound stimulation at 1000 Hz, the sound intensity at 90 dB showed the clearest waveform of cVEMP (Additional file 1: Figure S5B). Also, sound stimulation at 1000 Hz and 90 dB showed a clearer waveform of cVEMP than that at 500 Hz or 90 dB (Additional file 1: Figure S6). Therefore, we used sound stimulation at 1000 Hz and 90 dB of sound intensity in this study. These results partially correspond to the results of a previous study showing that sound stimulation at 1000 Hz is more sensitive for the detection of cVEMP than that at 500 Hz [28]. In this study, we also showed that the side on which intratympanic injection of gentamicin was performed had impaired cVEMP with delayed latency of more than 9 ms and decreased amplitude of less than 5 μV [i.e., out of the ranges of normal latency (6–9 ms) and normal amplitude (5–20 μV)] and impaired morphology of vestibular hair cells, while the control side showed normal cVEMP and intact morphology of vestibular hair cells (Additional file 1: Figure S7). These results are similar to the results of a previous study [11]. In this study, the latencies and amplitudes of normal cVEMPs in mice (6–9 ms and 5–20 μV, respectively) are similar to those in previous studies with guinea pigs and mice [12, 23, 24], while the latencies and amplitudes in mice are faster and smaller, respectively, than those in humans presumably because of the short conduction time and thinner muscle fibers in smaller animals. This interpretation for the differences of cVEMPs in rodents and humans was also given in a previous report [12].

There were significant correlations of cVEMP with imbalance behaviors in mice orally exposed to IDPN in this study. The scores of beam and air-righting reflex tests had significant correlations with amplitude and latency of cVEMP. In previous studies, the amplitude and latency of cVEMP were shown to reflect the activity and conduction velocity of the vestibular nucleus, respectively, in humans [33, 34]. Beam and air-righting reflex tests have been shown to reflect vestibulomotor function and vestibular reflex, respectively, in experimental animals [35]. This study showed a significant correlation of the number of hair cells in the saccule with cVEMP amplitude. Thus, our results demonstrated that the amplitude of cVEMP significantly correlates with vestibular activities derived from the saccule in a mouse model of vestibular disorder. In previous studies, intraperitoneal injection of IDPN in rats caused morphological damage of the crista ampullaris in the semicircular canals, resulting in imbalance behaviors in horizontal motor activity tests and an air-righting reflex test [16, 36–38]. In this study, there were significant correlations of the numbers of hair cells in the utricle and the cupula with cVEMP amplitude presumably due to the widespread distribution of IDPN in inner ears. The number of hair cells in the utricle was also correlated with cVEMP latency. The canal function has been determined by the vestibule-ocular reflex (VOR) test [39]. Therefore, it would be worthwhile to determine the correlation of VOR with scores of behavior tests in mice exposed to IDPN.

In this study, the correlation coefficient between the beam test score and cVEMP amplitude was larger than that between the air-righting reflex test score and cVEMP amplitude, while the correlation coefficient between the beam test score and cVEMP latency was less than that between the air-righting reflex test score and cVEMP latency. Therefore, it is possible that the beam test score has a stronger association than the air-righting reflex test score with the activity of the vestibular nucleus, whereas the air-righting reflex test score has a stronger association with a conduction velocity of the vestibular nucleus. On the other hand, the rotarod test score was correlated with the amplitude of cVEMP but not with the latency of cVEMP in this study. The rotarod test has been used for determination of vestibular function [17, 19] and also other physiological functions including motor skill learning [40–42]. Therefore, it is unlikely that cVEMP latency is associated with physiological functions other than the vestibular systems. In this study, swim and footprint tests were not performed, although these tests have been examined to determine the balance in previous studies [6, 43]. Additional study is needed to determine correlations of cVEMP latency with scores of swim and footprint tests.

In this study, correlations among balance-related behaviors in the IDPN and control groups were analyzed (Additional file 1: Figure S8) since there is no direct information about the imbalance in IDPN-exposed mice determined by a beam test. The scores of the beam test had significant correlations with scores of the rotarod test (*p* = 0.0001, *r* = − 0.6423) and air-righting reflex test (*p* < 0.0001, *r* = 0.6861) (Additional file 1: Figure S8A, C). Thus, these results suggest that imbalance in mice exposed to IDPN can be determined by using a beam test. In this study, the correlations of cVEMP with balance-related behaviors were determined at 3–4 days after oral administration of IDPN at the high dose of 28 mmol/kg to mice at 4 weeks of age, since toxicity in mice exposed to IDPN at 3 weeks of age has been shown to be less than that in aged mice [44]. In fact, all of the IDPN-administered mice survived for at least for 1 month in this study, whereas IDPN-administered mice aged 8 to 10 weeks that were orally administered IDPN at a dose of 28 mmol/kg were dead at 1 week after administration in a previous study [18]. In a previous study using scanning electron microscopy, it was shown that most of the hair bundles in the utricle and saccule were lost in guinea pigs at 4–6 weeks after oral administration of IDPN at 3.2 mmol/kg [18], while the numbers of hair bundles in the utricle and saccule detected by phalloidin staining were significantly decreased in the IDPN group at 3–4 days after oral administration of IDPN at 28 mmol/kg in this study. Thus, it is likely that defects similar to those in the exposed guinea pigs occurred in the mice exposed to IDPN, although the dose and time after administration were different. It will be necessary to determine the correlations in mice exposed to IDPN at lower doses and different days after administration.

Other mouse models of vestibular disorder including Ames waltzer mice [45] and Slitrk6-deficient mice [46] have been used in previous studies. Therefore, it is also necessary to determine the correlations in other genetic mouse models. In addition, chronic exposure to low-frequency noise (LFN) has been shown to affect the vestibule in mice [47]. LFN is known to be generated from many devices including industrial machines in daily and occupational environments [47]. Exposure to toxic elements has also been shown to affect our health including balance [48, 49]. Thus, the imbalance caused by environmental factors is one of the serious problems in environmental and occupational health. However, there is very limited information about the prevention of imbalance caused by environmental factors because of the limited number of methods for evaluation of the vestibular function in experimental animals. In particular, information about cVEMP in mouse models of vestibular disorders is very limited compared to information about ABR, although cVEMP is clinically used to determine vestibular function in humans. This experimental study demonstrated the usefulness of cVEMP for determination of vestibular function in a mouse model of vestibular disorder induced by IDPN. Further study is needed to determine the correlation between balance determined by behavior tests and cVEMP in other mouse models of vestibular diseases caused by environmental factors in order to develop new preventive and therapeutic strategies against imbalance in humans.

## Conclusion

In conclusion, this study demonstrated for the first time correlations of cVEMP with imbalance determined by behavior tests in a mouse model of vestibular disorder.

## Additional file

Additional file 1: **Figure S1.** Typical waveforms of cVEMP with head tilt and the background. **Figure S2.** Vestibular hair cell loss in the saccule and utricle of mice treated with IDPN. **Figure S3.** Amplitude of preceding negative wave and amplitude of p1-n1.**Figure S4.** Typical ABR waveforms of the control group (A) and the IDPN group (B) at 20–80 dB SPL of 4 kHz sound are presented at the same scales. **Figure S5.** cVEMP recording in normal mice under anesthesia or under wakefulness. **Figure S6.** Typical waveforms of cVEMP elicited by sound stimulation of 500 Hz and 1000 Hz. **Figure S7.** Unilateral cVEMP recording and immunohistostaining of hair cells in the otoconia and cupula from each ear of gentamicin-treated mice. **Figure S8.** Correlations between scores of the behavior tests. (PDF 1630 kb)

## Data Availability

The datasets used analyzed during the current study are available from the corresponding author on reasonable request.

## Abbreviations

EMG: Electromyography
IDPN: 3,3′-Iminodipropiontrile
VEMP: Vestibular evoked myogenic potential
