# Supplementary material for: cVEMP correlated with imbalance in a mouse model of vestibular disorder
Source: Environ Health Prev Med. 2019 Jun 1;24:39. doi: 10.1186/s12199-019-0794-8 (PMC6545207; doi:10.1186/s12199-019-0794-8)
Supplement: Supplementary file 1 — Figure S1. Typical waveforms of cVEMP with head tilt and the background. Figure S2. Vestibular hair cell loss in the saccule and utricle of mice treated with IDPN. Figure S3. Amplitude of preceding negative wave and amplitude of p1-n1.Figure S4. Typical ABR waveforms of the control group (A) and the IDPN group (B) at 20–80 dB SPL of 4 kHz sound are presented at the same scales. Figure S5. cVEMP recording in normal mice under anesthesia or under wakefulness. Figure S6. Typical waveforms of cVEMP elicited by sound stimulation of 500 Hz and 1000 Hz. Figure S7. Unilateral cVEMP recording and immunohistostaining of hair cells in the otoconia and cupula from each ear of gentamicin-treated mice. Figure S8. Correlations between scores of the behavior tests. (PDF 1630 kb) [file 12199_2019_794_MOESM1_ESM.pdf]

Supplementary Material

cVEMP correlated with imbalance in a mouse model of vestibular disorder

Reina Negishi-Oshino, Nobutaka Ohgami, Thangchao He, Kyoko Ohgami, Xiang Li and Masashi Kato*

Correspondence: Masashi Kato, M.D., Ph.D., e-mail: katomasa@med.nagoya-u.ac.jp

Additional file 1

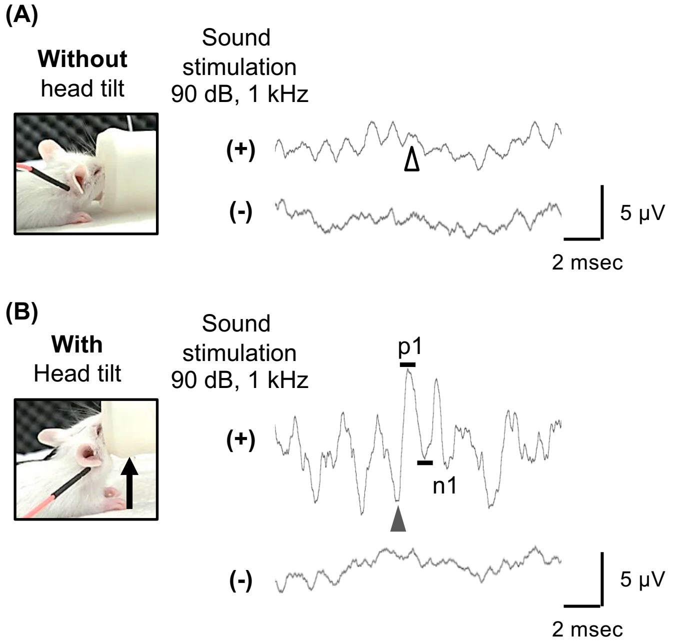

Figure S1. Typical waveforms of cVEMP with head tilt and the background.
Typical waveforms of cVEMP in electromyography (EMG) and the settings without **(A)** and with head tilt **(B)** under the conditions of sound stimulation at 1000 Hz and 90 dB SPL (+, upper waves) and no stimulation (-, lower waves) are shown. During cVEMP recording, the animal's neck was stretched by lifting up the inhalation mask with a polypropylene string to induce tonus of the sternocleidomastoid muscle. The background is shown in **(A)** with a white arrowhead. The inhalation mask was set at 2 cm in height from the ground as indicated by a blue arrow in the picture of **(B)**.

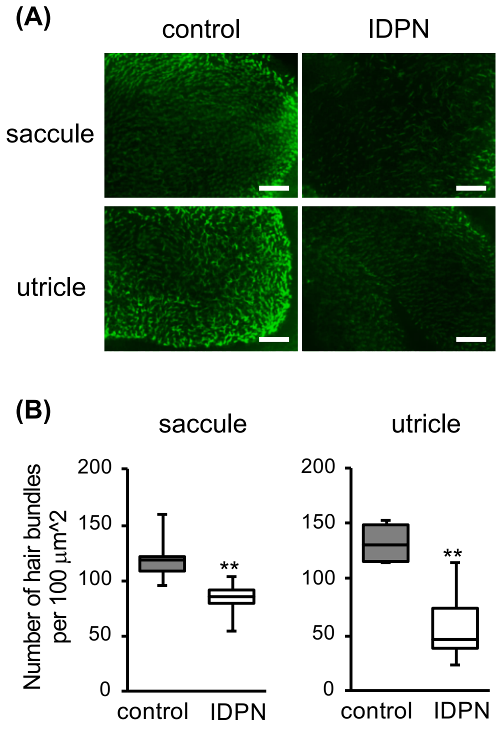

Figure S2. Vestibular hair cell loss in the saccule and utricle of mice treated with IDPN. **(A)** After administration of IDPN, hair bundles were stained with FITC-labeled phalloidin. Equivalent positions in the saccule (upper panels) and the utricle (lower panels) from mice in the control group (left panels) and mice in the exposure group (right panels) are shown with scale bars (50 µm). **(B)** Numbers of hair bundles per 100 μm^2 (means ± SD) in the saccule (left box plot) and the utricle (right box plot) from three mice in the control group (gray) and three mice in the IDPN group (white) are shown. Significant differences (**p < 0.01) between the two groups were analyzed by Welch's t-test.

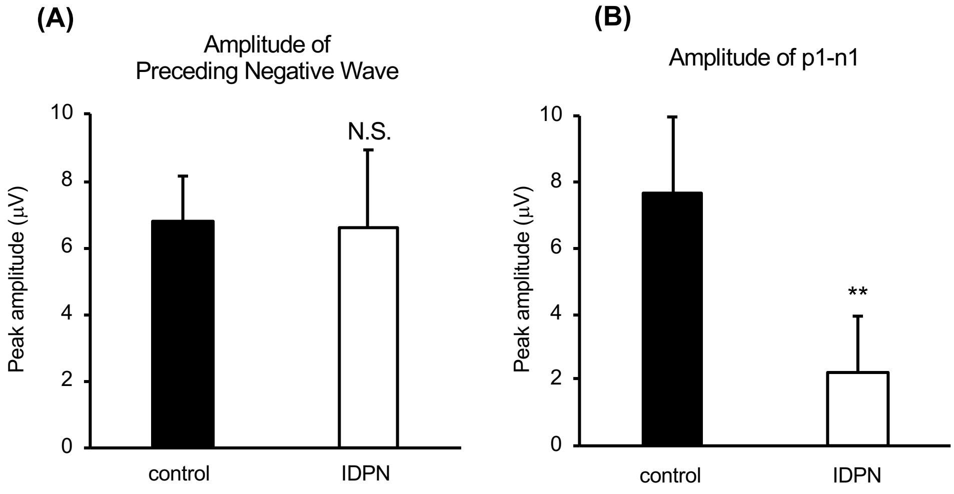

Figure S3. Amplitude of preceding negative wave and amplitude of p1-n1.

Amplitude of the preceding negative wave **(A)** and amplitude of p1-n1 **(B)** in the control group (black bars) and IDPN group (white bars) are shown. Significant differences (**p < 0.01) between the two groups were analyzed by Welch's t-test. N.S. indicates no significant difference.

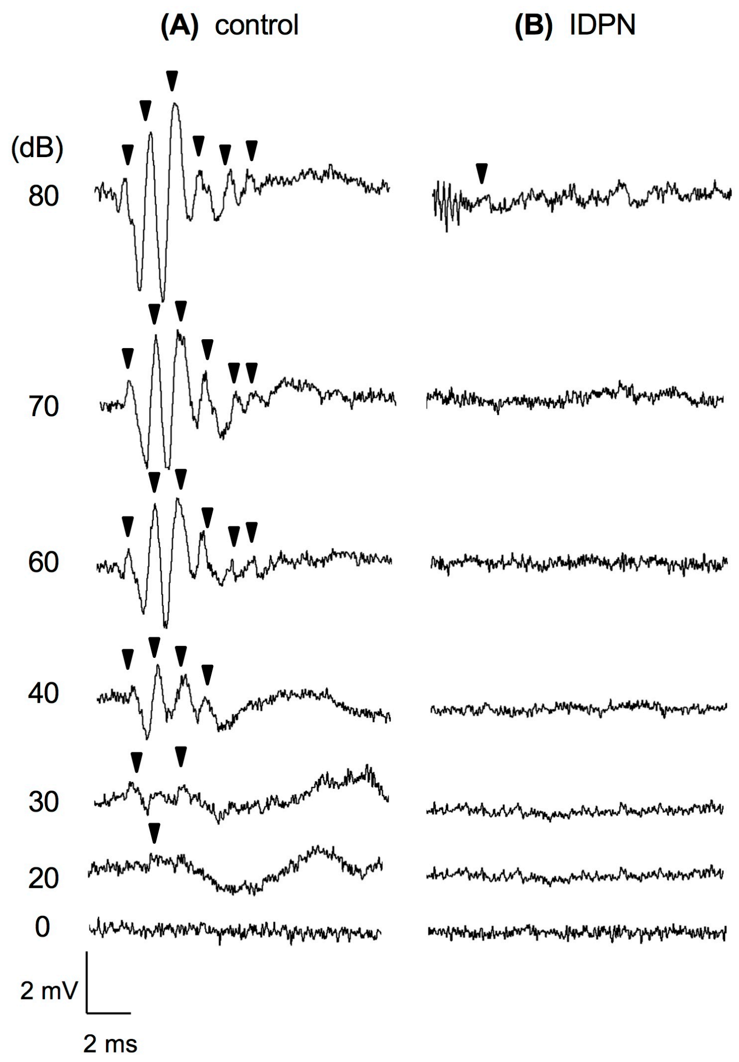

Figure S4. Typical ABR waveforms of the control group **(A)** and the IDPN group **(B)** at 20-80 dB SPL of 4 kHz sound are presented at the same scales. The peaks of ABR waves are indicated by arrowheads.

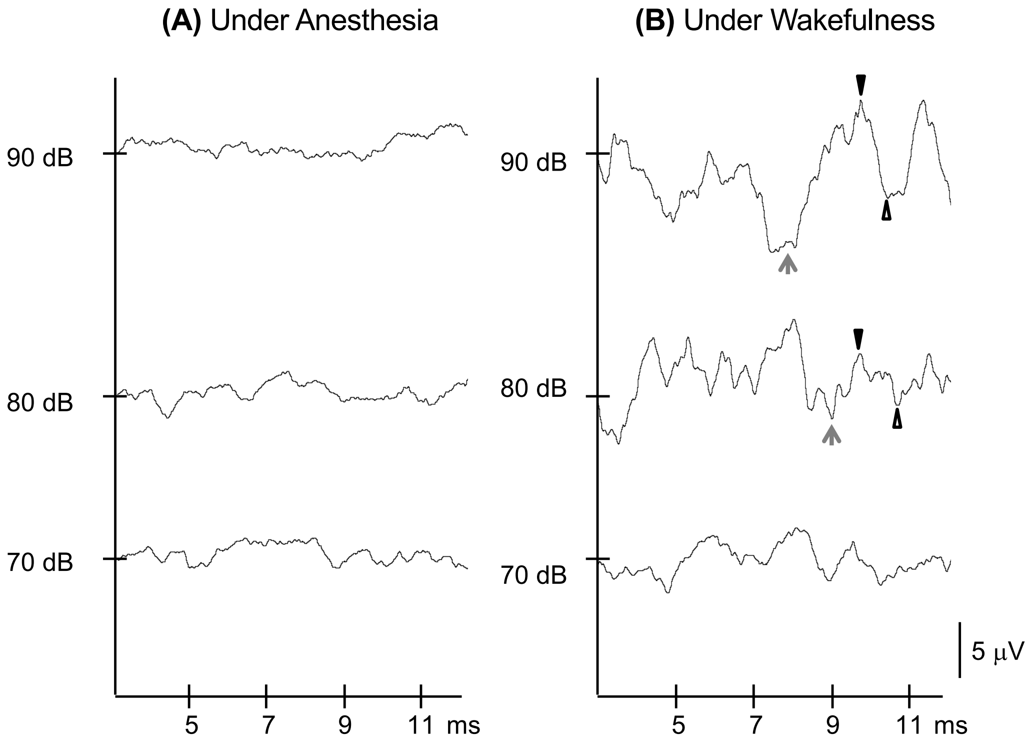

Figure S5. cVEMP recording in normal mice under anesthesia or under wakefulness. Typical waveforms of cVEMP were elicited by 1000 Hz of sound stimulation at 70-90 dB SPL. **(A, B)** cVEMP recorded with mice **(A)** under anesthesia and **(B)** under wakefulness (preceding negative wave, gray arrow; positive peak, black arrowheads; negative peak, white arrowheads) under the conditions of sound stimulation at 1000 Hz and 90 dB SPL (upper waves), 80 dB SPL (middle waves), and 70 dB SPL (lower waves). During cVEMP recording, the animal's neck was stretched by lifting up the inhalation mask with a polypropylene string to induce tonus of the sternocleidomastoid muscle.

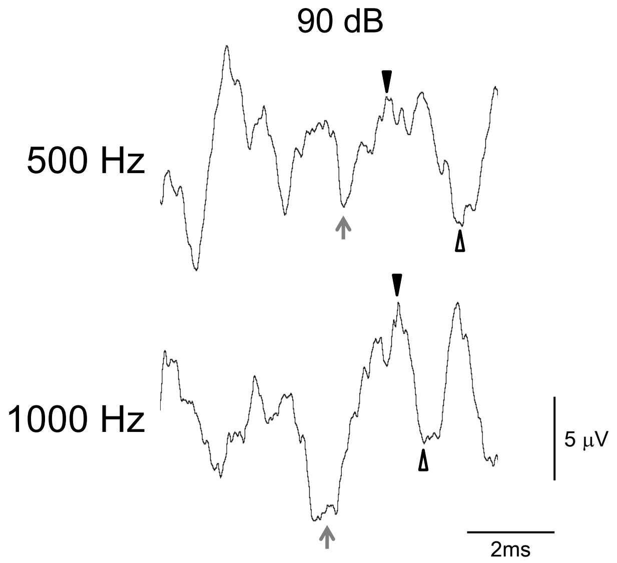

Figure S6. Typical waveforms of cVEMP elicited by sound stimulation of 500 Hz and 1000 Hz. cVEMPs were recorded in normal mice under the conditions of sound stimulation of 500 Hz (upper wave) and 1000 Hz (lower wave) at 90 dB SPL (preceding negative wave, gray arrow; positive peak, black arrowheads; negative peak, white arrowheads). During cVEMP recording, the animal's neck was stretched by lifting up the inhalation mask with a polypropylene string to induce tonus of the sternocleidomastoid muscle.

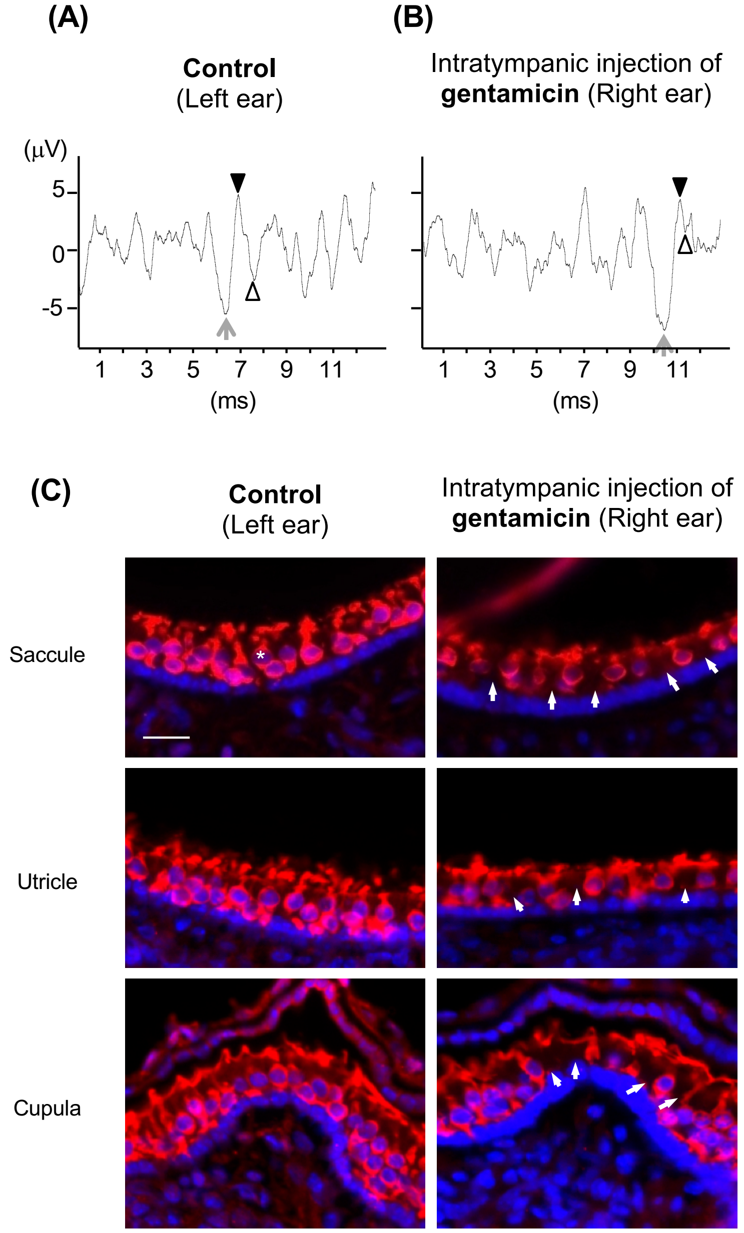

Figure S7. Unilateral cVEMP recording and immunohistostaining of hair cells in the otoconia and cupula from each ear of gentamicin-treated mice.

(A, B) cVEMP waveforms from **(A)** the left ear as a control and **(B)** the right ear treated with gentamicin (preceding negative wave, gray arrow; positive peak, black arrowheads; negative peak, white arrowheads). **(C)** Immunohistostaining of hair cells in the saccule (upper panels), utricle (middle panels), and cupula (lower panels) from the left ear as a control (left panels) and the right ear treated with gentamicin (right panels). Hair cells stained with anti-myosin-VIIa antibody are colored red and DAPI for counter staining is colored blue. An asterisk (upper left panel) shows an example of normal hair cells, while arrows indicate impaired hair cells (right panels). Scale bar: 20 μm.

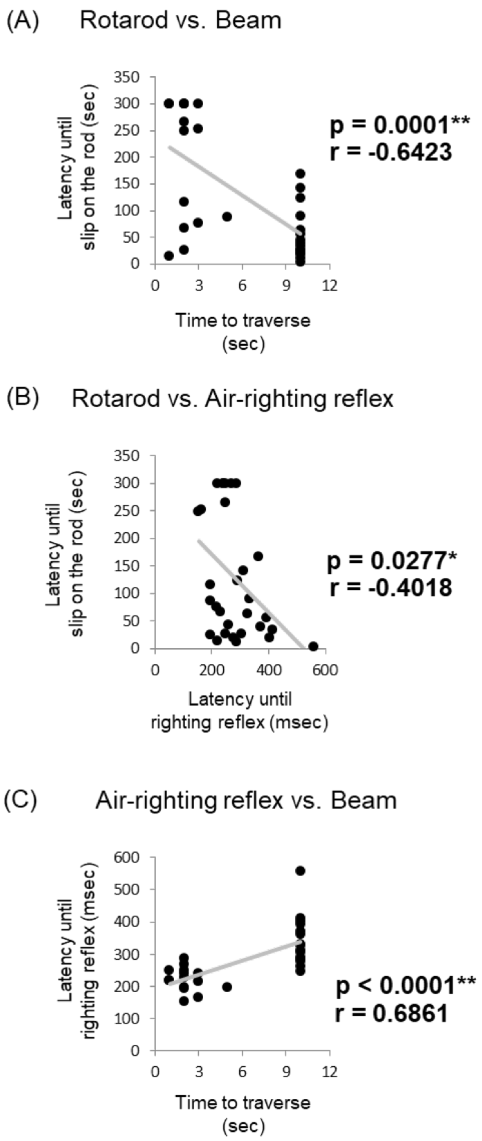

Figure S8. Correlations between scores of the behavior tests.

Pearson's correlation coefficient and p-value (**$p < 0.01$; *$p < 0.05$) were determined between scores of **(A)** the rotarod test and beam crossing test, **(B)** rotarod test and air-righting reflex test and **(C)** air-righting reflex test and beam crossing test. Triplicated measurements for each test with a total of ten mice including five mice for the control group and five mice for the IDPN group were performed.
